# VPAC1 receptor expression in peripheral blood mononuclear cells in a human endotoxemia model

**DOI:** 10.1186/1479-5876-11-117

**Published:** 2013-05-07

**Authors:** Angela Storka, Bernhard Burian, Gerhard Führlinger, Breanna Clive, Terri Sun, Richard Crevenna, Andrea Gsur, Wilhelm Mosgöller, Michael Wolzt

**Affiliations:** 1 Department of Clinical Pharmacology, Medical University of Vienna, Währinger Gürtel 18-20, Vienna, 1090, Austria; 2 Institute of Cancer Research, Department of Medicine I, Medical University of Vienna, Währinger Gürtel 18-20, Vienna, 1090, Austria; 3 Department of Pneumology, Medical University of Vienna, Währinger Gürtel 18-20, Vienna, 1090, Austria; 4 Department of Physical Medicine and Rehabilitation, Medical University of Vienna, Währinger Gürtel 18-20, Vienna, 1090, Austria

## Abstract

**Background:**

Vasoactive intestinal peptide (VIP) exerts immune-modulatory actions mainly via VPAC1 receptor stimulation. VPAC1 may be a treatment target of inflammatory diseases, but little is known about the receptor expression profile in immune-competent cells *in vivo*.

**Material and methods:**

20 male healthy subjects received a single intravenous bolus of 2ng/kg body weight Escherichia coli endotoxin (LPS). Receptor status was evaluated in peripherial blood cells before and 3, 6 and 24 h after LPS by FACS analysis and q-PCR. VIP plasma concentrations were measured by ELISA.

**Results:**

Granulocytes accounted for 51% of leukocytes at baseline and 58 ± 37% were positive for VPAC1. The granulocyte population increased 2.6 fold after LPS, and a transient down-regulation of VPAC1 to 28 ± 23% was noted at 3 h (p < 0.001), which returned to baseline at 24 hours. Baseline VPAC1 expression was low in lymphocytes (6.3 ± 3.2%) and monocytes (11 ± 9.6%). In these cells, LPS up-regulated VPAC1 at 6 h (13.2 ± 4.9%, p < 0.001) and 24 h (31.6 ± 20.5%, p = 0.001), respectively. Consistent changes were noted for the VIP-receptors VPAC2 and PAC1. VPAC1, VPAC2 and PAC1 mRNA levels were unchanged in peripheral blood mononuclear cells (PBMC). VIP plasma concentration increased from 0.5 ± 0.3 ng/ml to 0.7 ± 0.4 ng/ml at 6 h after LPS (p < 0.05) and returned to baseline within 24 h.

**Conclusion:**

The time profile of VPAC receptor expression differs in granulocytes, monocytes and lymphocytes after LPS challenge in humans. Changes in circulating VIP concentrations may reflect innate immune responses.

## Background

Vasoactive intestinal peptide (VIP) has a broad range of biological actions such as dilatation of smooth muscle cells including broncho- and vasodilation, insulin releasing properties, influence on intestinal motility, and neuroprotective capacities [1-8]. VIP is also known as a potent immunomodulator [9-11]. Down-regulation of tumor necrosis factor α (TNFα) [12], IL-6 [13], IL-8, IL-12 [14], inducible nitric oxide synthase (i-NOS) [15], and enhanced production of anti-inflammatory cytokines such as IL-10 and IL-1RN [16] have all been described to contribute to the direct anti-inflammatory action of VIP.

These functions are mediated via three different G-protein coupled receptors, VPAC1, VPAC2 and PAC1, which are localized on immune-competent cells and on other mesenchymal and endothelial tissues in humans [17-20]. For the anti-inflammatory signaling, VPAC1 has been identified as the most important receptor [21].

Since VIP and VPAC-1 receptor agonists appear to affect synthesis and release of multiple cytokines, they might provide an efficient therapeutic alternative to the use of specific cytokine antibodies or antagonists as immune modulatory agents. Previous reports showed that administration of VIP attenuates the deleterious consequences of septic shock and has been successfully used in various animal models of rheumatoid arthritis, multiple sclerosis and inflammatory bowel disease [22-26]. However, studies characterizing the expression of VPAC1 receptors on immune-competent cells and their regulation following immunological stimulation in humans are not available.

This study has therefore addressed VPAC receptor expression profiles in peripheral venous blood following systemic LPS administration in healthy human subjects. Small doses of intravenously administered LPS cause an acute inflammatory response, qualitatively similar to that occurring during the early stages of sepsis. Transient changes in systemic hemodynamics, endothelial function, permeability, pulmonary gas exchange and ventricular function can be induced within 3 hours of intravenous administration of LPS to healthy subjects. The results of this study may help to clarify to which extend the VIP receptors are involved in the systemic inflammatory response in humans.

## Material and methods

The study was approved by the Ethics Committee of the Medical University Vienna (EK 725/2007) and conforms to the principles outlined in the Declaration of Helsinki, including current revisions and the Good Clinical Practice guidelines. 20 healthy male subjects aged from 18 to 45 years were included. Following a complete health examination, including physical examination, electrocardiogram, and laboratory screening subjects received intravenously 2 ng/kg body weight of *E. coli* endotoxin (U.S. Standard Reference Endotoxin; NIH-CC, Bethesda, MD) lipopoysaccharide (LPS) within a period of 3 minutes to induce a systemic inflammatory response. Venous blood samples were collected at baseline, 3 hours, 6 hours and 24 hours after LPS administration for peripheral blood mononuclear cells (PBMC) isolation and VIP serum level quantification. For technical reasons white blood cell count was only available from 10 subjects.

### Receptor expression in peripheral venous blood

To examine the expression of VPAC1, VPAC2 and PAC1 on different blood cells, FACS analysis was performed from whole blood. As primary antibodies monoclonal mouse anti VPAC1, mouse anti VPAC2 and mouse anti PAC1 antibodies (abcam Antibody Solutions, Cambridge, UK) were used at 1:200 dilution. Following an incubation period of 20 min at 4°C blood cells were washed with FACS buffer (phosphate buffered saline supplemented with 8 mM EDTA, 0.2% bovine serum albumin and 7.6 mM sodium azide). The secondary antibody (FITC labeled goat anti mouse antibody, Bethyl, Montgomery TX, USA) was allowed to incubate for 20 min at 4°C. The different cell populations were distinguished in the forward and side scatter and confirmed by double staining for monocytes or lymphocytes, respectively (Online Annex Additional file 1: Figure S2, Additional file 2: Figure S3, Additional file 3: Figure S4). Monocytes were detected using an anti-CD14 antibody (CD14-RPE, DAKO, Glostrup, Denmark) and lymphocytes with an anti-CD3 antibody (CD3-PE, Becton Dickinson, San Diego CA, USA). Control stainings were performed with isotype-matched antibodies. Following antibody incubation the cells were washed using FACS buffer and erythrocytes were lysed in 1.55 M NH_4_Cl, 100 mM KHCO_3_ and 1 mM EDTA. As negative control, MOLT-4cells (ATCC, Middlesex, UK) which are known not to express VPAC-1, were used.

Acquisitation was performed on a Becton Dickinson FACScan flow cytometer (Becton Dickinson, San Diego CA USA) by analysing 100.000 to 300.000 events per sample.

### VIP plasma concentration

After blood collection in EDTA buffered tubes, 50 μl Trasylol (Bayer Health Care Pharmaceuticals, Barmen, Germany) was added and centrifuged at 2000 g for 10 minutes at 4°C immediately to avoid degradation. EDTA plasma was frozen at -80°C. Peptides were extracted from the plasma using C-18 sept colums (containing 200 mg of C18), Buffer A and Buffer B according the protocol (Phoenix Pharmaceuticals Inc., Karlsruhe, Germany) and VIP levels measured using a commercial available enzyme linked immunoassay (Phoenix Pharmaceuticals Inc., Karlsruhe, Germany) following the manufacturer´s protocol within 1 month after sample collection. The enzyme linked immunoassay quantifies concentrations between 0.12-25 ng/ml.

### VIP receptor mRNA in PBMC

Venous peripheral blood mononuclear cells (PBMC) were harvested using Ficoll density gradient centrifugation. Ficoll-Paque plus (GE Healthcare Life Sciences, Chalfont St Giles, GB) was overlayed with 5 ml EDTA blood and centrifuged for 20 minutes at 500 g at room temperature. Cells were then collected, washed twice with phosphate buffered saline (PBS) and harvested for PCR analysis.

Total RNA was isolated from PBMC using the Trizol reagent (Invitrogen, California US) according to the manufacturer’s instruction. Integrity of RNA was verified by gel electrophoresis. An aliquot of 2 μg RNA was reverse transcribed into first strand cDNA with RevertAid™ First Strand cDNA Synthesis Kit (Fermentas, Massachusetts, USA) using random hexamer primer in compliance with the standard protocol for PCR amplification. qPCR was carried out using TaqMan® Gene Expression Master Mix (Applied Biosystems, California, USA), TaqMan® Gene Expression Assays (Applied Biosystems, California US) and 1ng cDNA in a 20 μl reaction mixture. The PCR was performed on the 7500 Fast Real-Time PCR System (Applied Biosystems, California USA) under following conditions: an initial incubation at 50°C for 20 s and 95°C for 10 min followed by 40 cycles of 95°C for 15 s, 54°C for 1 min.

Threshold cycle-values (Ct) for the VPAC1 (NM_001251882.1, NM_001251883.1, NM_001251884.1, NM_001251885.1, NM_004624.3), VPAC2 (NM_003382.4) and PAC1 (NM_001099733.1, NM_001117.3) genes and the B2M housekeeping gene were determined in triplicates. Relative quantification of RNA was calculated by ΔΔ Ct method. Omission of cDNA was used as negative control.

### Statistical analysis

Datasets were described using descriptive statistics as means ± SD. Data distribution was skewed and non-parametric tests were performed accordingly using SPSS® software (IBM, NY, USA). After assessing Friedman-ANOVA analysis the Wilcoxon matched-pairs test was used to compare groups. A p-value of 0.05 was considered statistically significant.

## Results

All participants (age: 29 ± 6 years, body mass index: 20.7-25.0 kg/m^2^) completed the study per protocol. As expected, systemic administration of LPS induced mild and transient flu-like symptoms. These included temporary feeling of illness, increase in body temperature, shivering, headache, myalgia, sickness or nausea. Systemic blood pressure was slightly reduced and heart rate increased after LPS (Table 1). Following LPS white blood cell count increased significantly at 3 h and 6 h and returned to baseline levels after 24 h (Table 2). Neutrophil counts increased after LPS administration up to 2.6-fold after 6 h and returned to baseline levels after 24 h. After 3 h and 6 h a decrease of monocyte and lymphocyte cell counts by 89% and 31%, and by 75% and 74%, respectively, was seen.

**Table 1:** Vital signs

**Time**	**Mean blood pressure (mmHg)**	**Heart rate ** **(bpm)**	**Body temperature (°C)**
**Baseline**	97 ± 9	73 ± 10	36.1 ± 0.4
**LPS 3 hours**	92 ± 10*	87 ± 11*	37.1 ± 0.5*
**LPS 6 hours**	93 ± 10*	86 ± 9*	36.9 ± 0.4*

Mean arterial blood pressure, heart rate and body temperature at baseline and following systemic LPS administration. Data are means ± SD (n = 20). Differences from baseline are indicated (*p < 0.001, Wilcoxon matched-pairs test).

**Table 2:** White blood cell count

**Time**	**Total WBC (G/l)**	**Granulocytes (G/l)**	**Monocytes (G/l)**	**Lymphocytes (G/l)**
**Baseline**	7.3 ± 1.4	3.9 ± 1.0	0.7 ± 0.2	2.4 ± 0.5
**LPS 3 hours**	9.8 ± 2.7*	9.0 ± 2.5*	0.1 ± 0.0*	0.6 ± 0.2*
**LPS 6 hours**	11.2 ± 1.4*	9.9 ± 1.1*	0.5 ± 0.2*	0.6 ± 0.2*
**LPS 24 hours**	6.4 ± 1.1	3.5 ± 0.8	0.6 ± 0.1	1.9 ± 0.4

White blood cell (WBC) count and subsets at baseline and 3 h, 6 h and 24 h after systemic LPS administration (mean ± SD, n = 10). Differences from baseline are indicated (*p < 0.001, Wilcoxon matched-pairs test).

### VPAC1 receptor expression

VPAC1 receptors expression was not detectable in MOLT-4 cells (data not shown), which were used as negative controls. The mean fluorescence intensity (MFI) reflecting the absolute receptor signal (receptor expression) in the different cell populations is summarized in Table 3. Individual values of VPAC-1 receptor expression in the different cell types are shown in Online Additional file 4: Figure S5.

**Table 3:** Mean fluorescence intensity of VPAC1, VPAC2 and PAC1

**Time**	**Granulocytes**	**Monocytes**	**Lymphocytes**
A - VPAC1			
**Baseline**	30.5 ± 16.5	19.9 ± 11.7	29.6 ± 11.7
**LPS 3 hours**	24.3 ± 9.3 *	19.9 ± 20.4	34.1 ± 19.0
**LPS 6 hours**	21.4 ± 9.9 *	15.3 ± 9 *	32.5 ± 17.3
**LPS 24 hours**	28.5 ± 16.1	18.2 ± 7.3	31.4 ± 23.7
B - VPAC2			
**Baseline**	19.9 ± 5.7	17.4 ± 7.6	23.5 ± 10.9
**LPS 3 hours**	17.4 ± 7.5	13.6 ± 6.3 *	20.7 ± 10.8
**LPS 6 hours**	35.7 ± 86.6 *	16.0 ± 9.2	32.4 ± 41.1
**LPS 24 hours**	18.3 ± 7.9	19.1 ± 12.7	21.1 ± 8.5 *
C - PAC1			
**Baseline**	17.4 ± 6.2	14.8 ± 5.3	21.6 ± 10.2
**LPS 3 hours**	16.7 ± 8.6	12.5 ± 10.3	15.7 ± 10.2
**LPS 6 hours**	21.8 ± 28.1	12.5 ± 9.6 *	17.6 ± 7.2
**LPS 24 hours**	17.4 ± 6.0	15.4 ± 5.8	15.7 ± 6.2

Mean fluorescence intensity values of VPAC1 (A), VPAC2 (B) and PAC1 (C) in granulocytes, monocytes, and lymphocytes. Values are expressed as mean ± SD (n = 20). Differences from baseline are indicated (*p < 0.005, Wilcoxon matched-pairs test).

### Granulocytes

**Figure 1:**
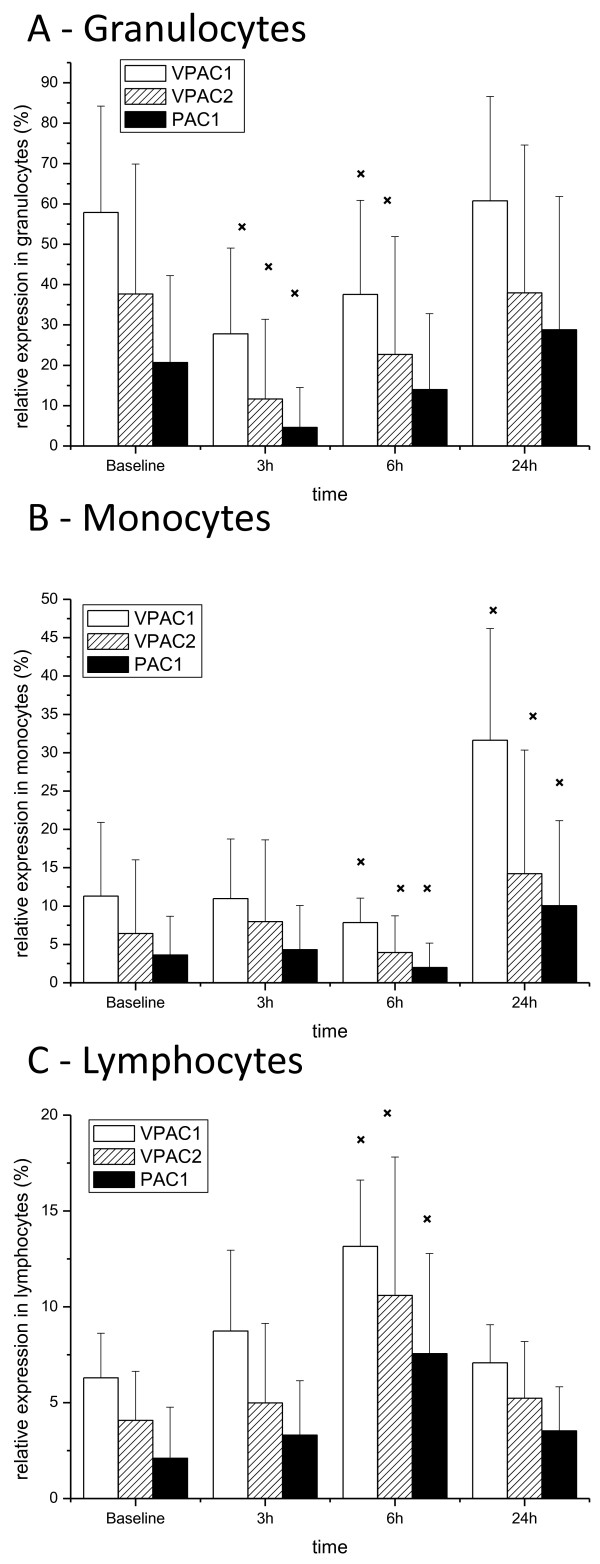
**VIP receptor expression after LPS stimulation.** Receptor expression after systemic LPS administration in **A**) granulocytes, **B**) monocytes, and **C**) lymphocytes. Data are presented as means ± SD, n = 20. Changes from baseline are indicated (*p < 0.001, Wilcoxon matched-pairs test).

At baseline 58 ± 37% of granulocytes were positive for VPAC1 receptors. Following LPS administration the number of VPAC-1 positive cells was lower after 3 hours and 6 hours compared to baseline (Figure 1A). This was paralleled by decreased MFI values (Table 3).

### Monocytes

The VPAC-1 expression profile was different in monocytes (Figure 1B). At baseline 11.3 ± 9.6% of monocytes were positive for VPAC1. LPS initially decreased and subsequently increased receptor expression to 7.8 ± 3.2% after 6 hours and to 31.6 ± 20.5% after 24 hours. This pattern was also detectable for MFI values (Table 3).

### Lymphocytes

In lymphocytes VPAC-1 receptor expression at baseline was 6.3 ± 3.2% (Figure 1C). LPS increased receptor expression to 8.7 ± 5.9% after 3 hours and to 13.2 ± 4.9% after 6 hours. MFI values were not significantly altered in lymphocytes after LPS (Table 3).

### VPAC1 mRNA expression

VPAC1 mRNA expression in PBMC was not changed after LPS administration (data not shown).

### VIP plasma levels

VIP plasma concentrations were 0.5 ± 0.3 ng/ml at baseline and increased to 0.6 ± 0.3ng/ml 3 hours and to 0.7 ± 0.4 ng/ml 6 hours after LPS (p < 0.05 versus baseline). 24 hours after LPS VIP plasma concentration was 0.5 ± 0.2 ng/ml.

### VPAC2 and PAC1 receptor and mRNA expression

Expression of VPAC2 receptors was substantially lower than that of VPAC1 in all three cell populations under study. A maximum of 5% of monocytes or lymphocytes stained positive for VPAC2 (Figure 1B,1C). Likewise, MFI values were lower compared to VPAC1.

After LPS administration the regulation pattern was consistent with that of VPAC1, with a downregulation of VPAC2 and PAC1 in granulocytes and an upregulation in monocytes and lymphocytes, respectively (Figure 1). Corresponding changes of MFI values were again detectable (Table 3). mRNA VPAC2 and PAC1 receptor expression was at the limit of quantification in PBMC and not altered by LPS.

## Discussion

The understanding of VPAC 1 receptor expression on immune-competent cells is a crucial step towards the development of VPAC agonists in human disease. This study demonstrates that in response to systemic low doses of LPS to healthy humans VPAC 1 receptor expression show a dynamic response in granulocytes, monocytes and lymphocytes. This LPS mediated effect is accompanied by a significant increase in VIP plasma concentrations.

The expression pattern of VPAC1 receptors is heterogeneous across different leukocyte populations. About 60% of granulocytes express VPAC1 under resting conditions, suggesting that these cells are particularly sensitive to alterations in VIP levels. Following LPS exposure granulocytes rapidly decrease the VPAC1 receptor density on their surface by approximately 50% within 3 hours. It is likely that receptor internalization as described by Langlet et al., [27] is responsible for this decrease in VPAC1 expression.

Granulocytes are recruited to infection sites and are of importance in the early microbial clearance [28,29]. This accumulation results in organ damage if unopposed pro-inflammatory signaling persists [30]. Thus, the ability to down-regulate leukocyte receptor activation after clearing the offending agent seems to be as essential as cell recruitment per se to prevent excessive tissue injury. The pattern of VPAC1 expression in granulocytes may therefore represent a regulator mechanism for granulocyte cell function.

The VPAC1 receptor expression profile was not consistent across leukocyte subtypes. Given that leukocyte count was significantly increased by LPS, the absolute reduction in monocyte and lymphocyte count indicates that their VPAC1 receptor - mediated effects presumably contribute to the net action of VIP to a small extent only. In the healthy subjects under study, baseline VPAC1 expression on monocytes and lymphocytes was low but increased following LPS by 2-3 – fold. Similar results have been shown in macrophages when stimulated with LPS in vitro [31]. Nevertheless, the upregulation detected in these cells may indicate a refinement in the immunological response to increased VIP concentrations.

The lack of changes in receptor mRNA in PBMC following LPS argues against a relevant receptor de novo synthesis in these cells. However, assessment of protein translation may not be indicative of receptor involvement in the immunomodulatory VIP-signalling since receptors are internalized after binding of their ligand VIP on the cell surface [32]. As suggested previoulsy [27], recycling and re-organization of receptors on the cellular surface may be responsible for the observed short-term effect of LPS in leukocytes.

Receptor stimulation is important for the pharmacodynamic action of VIP. In our model of endotoxemia we found a transient increase in VIP plasma concentrations. This is consistent with findings in septic patients, where an acute initial increase in VIP serum levels has been reported [33]. In contrast, decreased VIP serum levels have been described in patients with chronic pulmonary hypertension, where VPAC2 receptors are up-regulated in the pulmonary vasculature [34]. Thus, interpretation of altered VIP serum concentration remains speculative when the receptor expression is unknown.

The factors which influence VIP receptor expression are largely unclear. The present study demonstrates that VPAC1, VPAC2 and PAC1 expression is regulated in leukocytes following systemic stimulation with LPS *in vivo*. In contrast to Lara-Martinez et al, VPAC2 was expressed on monocytes [35]. However, VPAC1 is the predominant receptor in leukocytes. This is consistent with *in vitro* experiments where LPS is a powerful trigger for VPAC1 receptor regulation [31]. Animal studies have demonstrated that selective activation of VPAC1 is more effective in controlling the immunological answer than VPAC2 agonists [21]. The dynamic receptor regulation pattern observed in this study also favours VPAC1 as principal mediator of the anti-inflammatory action of VIP. Thus selective activation of VPAC1 may represent an efficacious clinical target to elicit anti-inflammatory actions without undesired side effects of simultaneous VPAC2 activation such as vasodilatation.

The present experiments were conducted employing *E. coli* endotoxin. It is possible that VPAC1 receptor regulation is variably sensitive to challenging agents from different bacteria, as previously described for cytokines such as TNF-α, INFγ or IL-10 [36]. The rapid serum-clearance of LPS indicates that secondary mediators may be involved in the delayed regulation of VPAC1 in PBC [37]. However, systemic LPS administration is more likely to activate immune-competent cells indirectly via systemic pro-inflammatory immunological mechanisms. The anti- inflammatory effect of VPAC1 agonists in clinical trials will therefore be influenced by the timing of administration and the type of infection and associated mechanisms of the innate immune system.

In summary our data support the concept that VPAC-1 is a promising immunomodulatory target receptor in humans.

### Grants

This project was supported and funded by “Medical Scientific Fund of the Mayor of the City of Vienna” (Project Number 09055).

## Competing interests

The authors declare that they have no competing interests.

## Authors’ contribution

MW and RC are responsible for the study design and trial conduct. AS and BB drafted the manuscript. AS collected clinical information and samples. BC, TS and GF conducted sample analyzes and prepared the manuscript. MW, AG and WM revised the manuscript. All authors read and approved the final manuscript.

## Supplementary Material

Additional file 1: Figure S2 Gating and IGg control. The different populations in PBC, granulocytes, lymphocytes and monocytes, have been gated using forward and side scatter (left). Additionally, isotype controls (upper right) and double stainings (lower right) have been performed. Granulocytes have been gated using forward and side scatter (lower left). Click here for file

Additional file 2: Figure S3 FACS dot blot of granulocytes. Granulocytes were gated in the forward and side scatter. The dot blot shows granulocytes stained with VPAC1-FITC at baseline (upper left). Following LPS administration a downregulation in VPAC1 receptor expression can be seen after 3 and 6 hours (upper right and lower left). Baseline levels are re-established after 24 hours (lower right). Click here for file

Additional file 3: Figure S4 VPAC1 in CD-14 positive cells. Monocytes were first gated using forward and side scatter. Additional control staining with CD-14PE was performed (A, left side). On the right side (B) CD-14 and VPAC1 positive monocytes are shown at baseline (upper left) and 3 hours (upper right), 6 hours (lower left) and 24 hours (lower right) after LPS. A clear increase in VPAC1 positive cells is seen 24 hours after LPS. Click here for file

Additional file 4: Figure S5. VPAC1 receptor expression in PBC (individual values). VPAC1 receptor expression for all subjects and blood cell populations are shown. (A, granulocytes; B, monocytes; C, lymphocytes). Click here for file
