# Supplementary figures and images for: VPAC1 receptor expression in peripheral blood mononuclear cells in a human endotoxemia model
Source: J Transl Med. 2013 May 7;11:117. doi: 10.1186/1479-5876-11-117 (PMC3651401; doi:10.1186/1479-5876-11-117)

## Slide 1
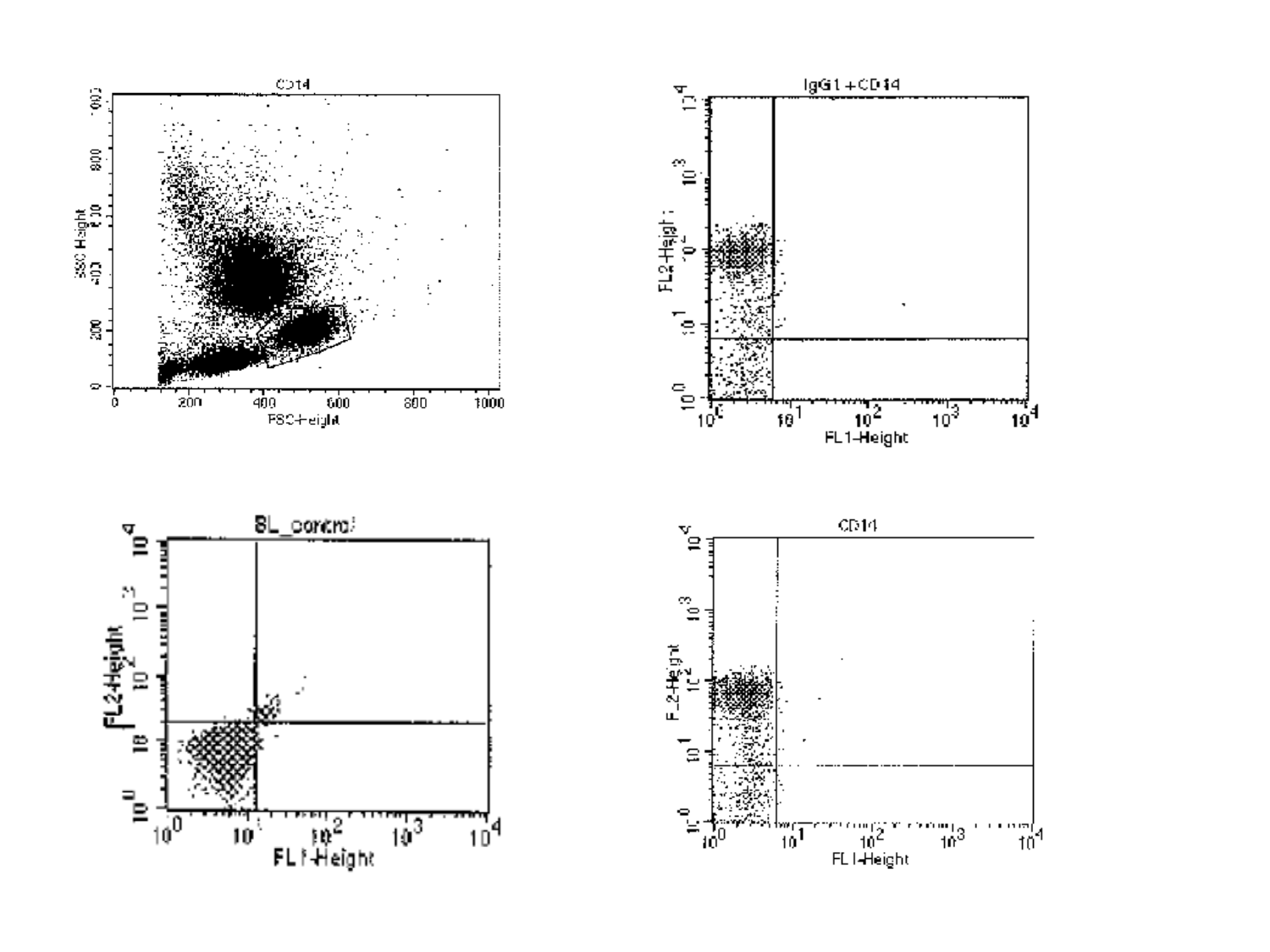

Supplement: Additional file 1: Figure S2 — Gating and IGg control. The different populations in PBC, granulocytes, lymphocytes and monocytes, have been gated using forward and side scatter (left). Additionally, isotype controls (upper right) and double stainings (lower right) have been performed. Granulocytes have been gated using forward and side scatter (lower left). [file 1479-5876-11-117-S1.ppt]

## Slide 1
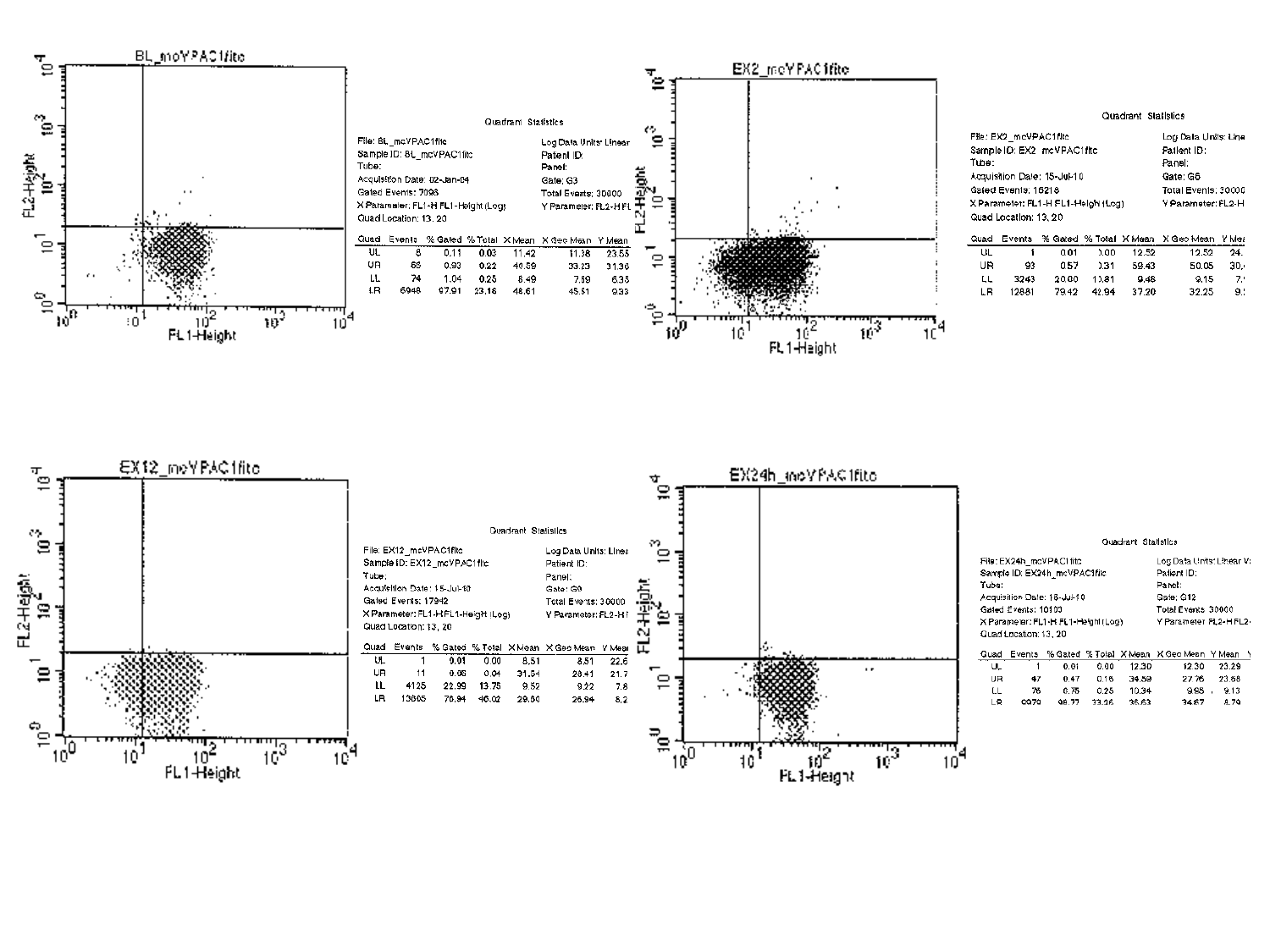

Supplement: Additional file 2: Figure S3 — FACS dot blot of granulocytes. Granulocytes were gated in the forward and side scatter. The dot blot shows granulocytes stained with VPAC1-FITC at baseline (upper left). Following LPS administration a downregulation in VPAC1 receptor expression can be seen after 3 and 6 hours (upper right and lower left). Baseline levels are re-established after 24 hours (lower right). [file 1479-5876-11-117-S2.pptx]

## Slide 1
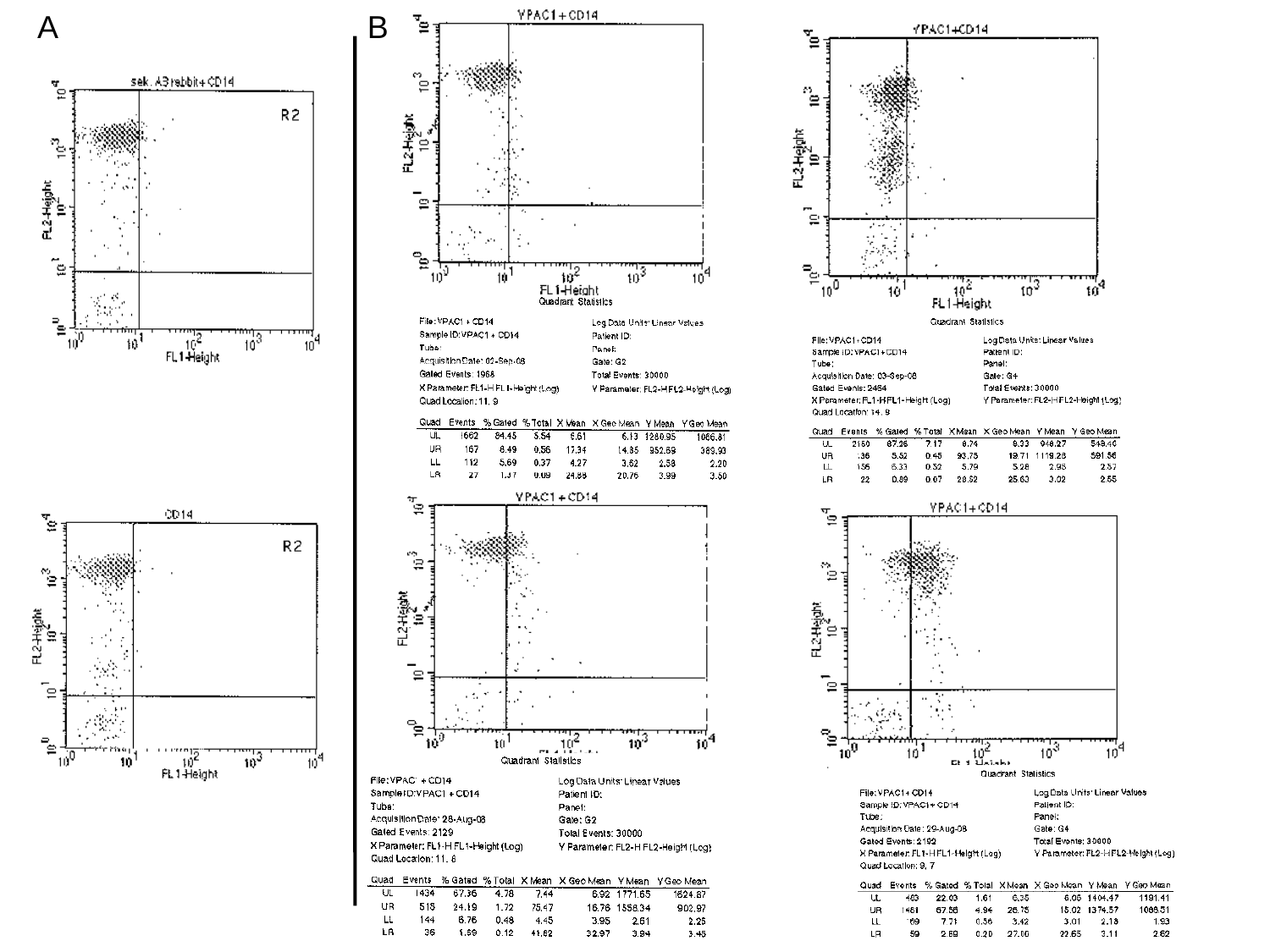

A
B

Supplement: Additional file 3: Figure S4 — VPAC1 in CD-14 positive cells. Monocytes were first gated using forward and side scatter. Additional control staining with CD-14PE was performed (A, left side). On the right side (B) CD-14 and VPAC1 positive monocytes are shown at baseline (upper left) and 3 hours (upper right), 6 hours (lower left) and 24 hours (lower right) after LPS. A clear increase in VPAC1 positive cells is seen 24 hours after LPS. [file 1479-5876-11-117-S3.ppt]

## Slide 1
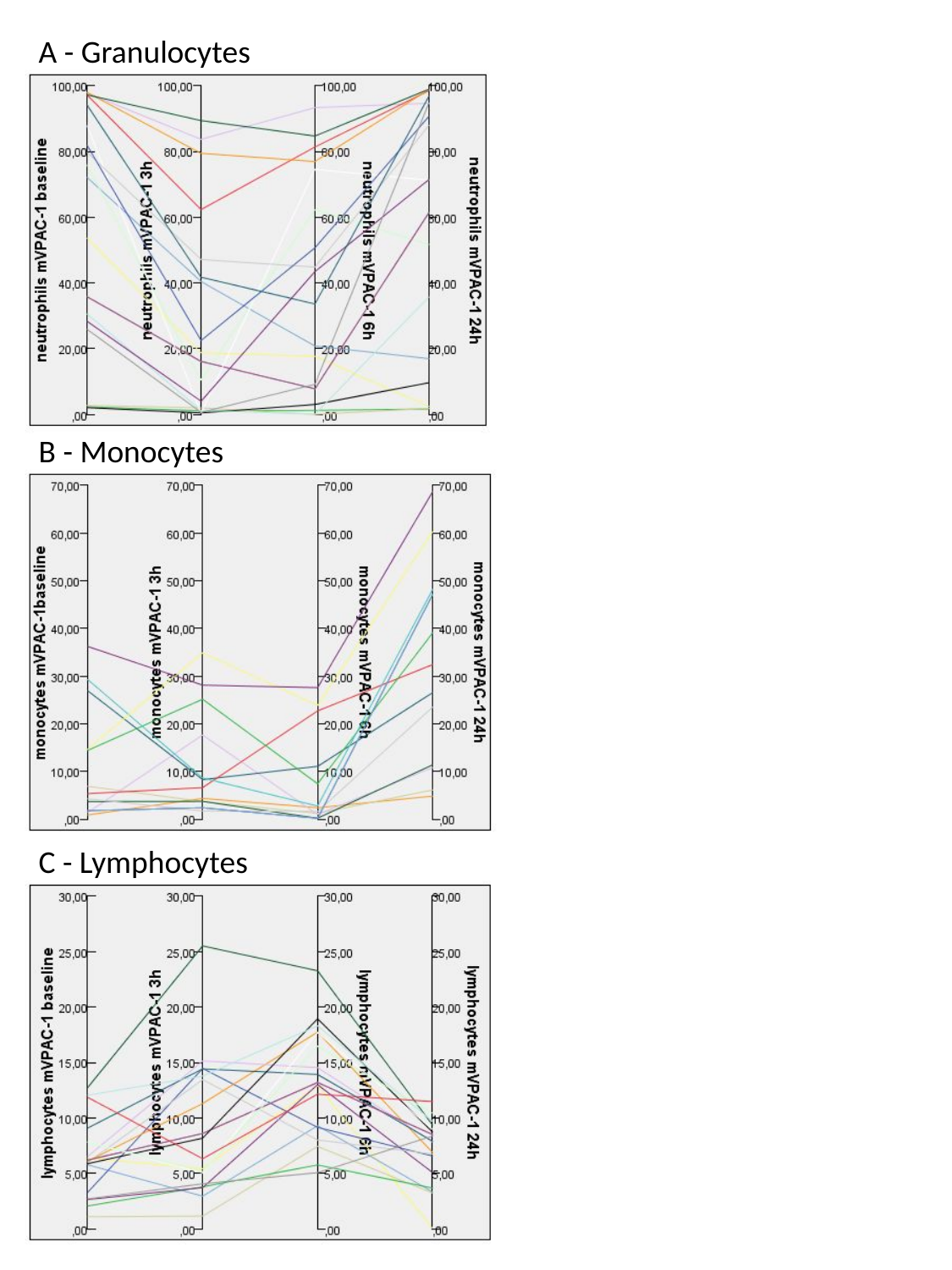

A - Granulocytes
B - Monocytes
C - Lymphocytes

Supplement: Additional file 4: Figure S5. — VPAC1 receptor expression in PBC (individual values). VPAC1 receptor expression for all subjects and blood cell populations are shown. (A, granulocytes; B, monocytes; C, lymphocytes). [file 1479-5876-11-117-S4.pptx]
